# Effect of valve spacing on peristaltic pumping

**DOI:** 10.1088/1748-3190/acbe85

**Published:** 2023-03-09

**Authors:** Ki Tae Wolf, Amir Poorghani, J Brandon Dixon, Alexander Alexeev

**Affiliations:** 1 George W. Woodruff School of Mechanical Engineering, Georgia Institute of Technology, Atlanta, GA 30332, United States of America; 2 Petit Institute for Bioengineering and Bioscience, Georgia Institute of Technology, Atlanta, GA 30332, United States of America; 3 Wallace H. Coulter Department of Biomedical Engineering, Georgia Institute of Technology, Atlanta, GA 30332, United States of America

**Keywords:** flow-vessel interaction, peristaltic micro pumping, elastic valves, valve spacing

## Abstract

Peristaltic fluid pumping due to a periodically propagating contraction wave in a vessel fitted with one-way elastic valves is investigated numerically. It is concluded that the valve spacing within the vessel relative to the contraction wavelength plays a critical role in providing efficient pumping. When the valve spacing does not match the wavelength, the valves open asynchronously and the volume of the vessel segments bounded by two consecutive valves changes periodically, thereby inducing volumetric fluid pumping. The volumetric pumping leads to higher pumping flowrate and efficiency against an adverse pressure gradient. The optimum pumping occurs when the ratio of valve spacing to contraction wavelength is about $2/3$. This pumping regime is characterized by a longer period during which the valves are open. The results are useful for further understanding the pumping features of lymphatic system and provide insight into the design of biomimetic pumping devices.

## 1. Introduction

The lymphatic vasculature consists of a complex network of vessels and valves which functions in parallel to the blood circulatory system [1, 2]. The lymphatic system plays a crucial role in maintaining homeostasis in the body by transporting the excess interstitial fluid, lipids, and immune cells from interstitial regions back to the cardiovascular system [1]. This excess fluid builds up in the interstitial region due to the leakage of fluid from the capillaries as determined by Starling’s Law [2]. The lymphatics maintain tissue fluid balance, while filtering lymph and providing a nexus for facilitating immune responses along the way. In addition, fatty acid from food is collected from enterocyte secretions in the interstitial spaces of the small intestine and transported via the lymphatic system [3]. Gathered lymph from the interstitial region is returned back to venous blood flow via subclavian veins at shoulder height in human [1].

Two principal types of lymphatic vessels are distinguished to dominate the fluid mechanics of lymph flow in the body: initial lymphatics and collecting lymphatics [4]. Initial lymphatic capillaries are blind-ended and responsible for the initial uptake of lymph from the interstitial region (some of $50\,\mu {\text{m}}$ in diameter) and connect upstream to collecting lymphatics [5]. The wall structure of initial lymphatics is comprised of thin single cell layer of endothelial cells with no basement membrane [6]. Lymph provided by initial lymphatics is fed into collecting lymphatic vessels. These vessels are subdivided into short segments between two consecutive one-way valves called the lymphangion [7]. Collecting mesenteric lymphatic vessels of rat average around $150\,\mu {\text{m}}$ in diameter [8]. Valves inside collecting lymphatics are spaced every $1$ to $3$ mm, which considering the diameter of the vessel is every 3–10 vessel diameters [9]. It is unknown what defines the spacing between consecutive valves in collecting lymphatics and to what extend the spacing is related to the vessel pumping performance.

The lymphatic system pumps lymph without a centralized pump as in the cardiovascular system. Pumping against an adverse gradient is achieved by either contraction from nearby skeletal muscles (extrinsic pump) [10] or through contraction of lymphatic vessels (intrinsic pump) [11]. The intrinsic pump is a result of periodic contractions of lymphangions driven through transient depolarization of lymphatic muscle cells. The contractions yield traveling waves propagating along the vessel and transporting the lymph against an adverse pressure gradient, whereas lymphatic valves prevent backflow enhancing pumping performance [12]. Ejection fraction of a lymphangion, defined as the ratio of lymph ejected out of the lymphangion during the contraction to the initial volume, can reach up to 80% [13].

Inequity between capillary filtration and lymph drainage results in accumulation of interstitial fluid and protein in the interstitial region, which when driven by lymphatic failure is referred to as lymphedema [14, 15]. Lymphedema can be caused by improperly functioning lymphatic vessels or valves. Currently lymphedema has no cure and is managed through medical compression garments, which only relieve the symptoms of the disease [2]. There are two main types of lymphedema: primary and secondary. Primary lymphedema develops due to underlying genetic mutations that lead to abnormal lymphatic development and defects in lymphatic valves [16]. Primary lymphedema is rare with estimated occurrence of 1 in 100 000 [17]. Approximately 99% of reported cases of lymphedema are secondary lymphedema [18]. Prevalence of secondary lymphedema is 1 in 1000 individuals, with patients being diagnosed mostly in the age range of 50–58 [19]. Secondary lymphedema occurs when previously normal lymphatics are obstructed or damaged by diseases, surgery, or radiation therapy [20].

Experiments show that pressure differences required for forward and backward flow in collecting lymphatics are roughly equal to $60$ Pa and $225$ Pa, respectively [11]. This difference points to the one-way nature of lymphatic valves. Collecting lymphatics can generate up to $60 \pm 20$ Pa of favorable pressure by intrinsic pumping mechanisms [21]. Experiments provide important insights into the lymphatic function, but also have significant limitations due to the small size and fragility of lymphatic vessels and valves [22]. Furthermore, the imaging of lymphatic valves is a challenging task [12]. These and other experimental constrains in studying the lymphatic system can be overcome using numerical models.

Intrinsic pumping mechanism of collecting lymphatics is associated with peristaltic vessel contractions. Theoretical and numerical models were developed to analyze peristaltic fluid flow and to identify parameters governing peristaltic pumping [23, 24]. Analytical models were suggested for peristaltic fluid pumping in two-dimensional channels with symmetric [25] and asymmetric [26, 27] wall motion. While these peristaltic flow models provide insights into the mechanisms of peristaltic pumping, these models disregard the effects of one-way elastic valves and, therefore, cannot be directly used to analyze lymphatic systems.

Lymphatic systems have also been modeled numerically using the lump parameter approach by solving differential equations fitted by experimental data [28, 29]. Lump parameter modeling was used for flow rate calculations and simulations of long chains of lymphatic segments [29, 30]. However, there is limited experimental data on lymphatic valve function under different flow conditions and adverse pressure gradients required in lump parameter models, thereby pointing to the need in developing more sophisticated fluid–structure interaction (FSI) models to rationalize lymphatic pumping [31].

FSI models enable more accurate modeling of fluid flow in lymphatic vessels with elastic valves. Due to the problem complexity, simplifying assumptions such as steady flow conditions and one-way coupling between the valves and the flow can be employed [31]. Even with these assumptions, the simulations were able to quantify the valve resistance for the forward and backward flows. More recently, a two-way fluid-solid interaction model was developed to investigate the intrinsic pumping in collecting lymphatics [32]. The model confirms experimentally reported hysteretic behavior of valves and reveals the formation of eddy structures in the flow near valves. The deformation of valve leaflets and vessel was investigated by considering hyperelastic material properties [33]. The simulations demonstrated that a lower pressure difference is required to open and close the valves when the valves or vessels are more flexible.

We have recently developed a three-dimensional, fully-coupled FSI model to investigate the behavior of compliant lymphatic valves in a static cylindrical vessel [34] and in a vessel that undergoes peristaltic contractions [35]. The simulations demonstrated the dependence of the lymphatic vessel pumping on the geometric features and mechanical properties of valve and the vessel contraction parameters such as contraction wave speed and amplitude. The results suggest that lymphatic vessels apply the maximum contraction work regardless of adverse pressure gradient, which is consistent with experimental data [36].

In this work, the effect of valve spacing on pumping performance is investigated. The flow performance metrics such as pumping rate, power, and efficiency are evaluated for different valve spacing and contraction wavelengths. The results show that a mismatch between inter-valve distance and the wavelength of the peristaltic wave can significantly increase pumping performance of the lymphatic vessel.

## 2. Methodology

An FSI solver is used for modeling viscous flow inside a contracting vessel with flexible valves [37, 38]. We model the three-dimensional fluid flow in lymphatic vessels using a lattice Boltzmann model (LBM) that recovers the Navier–Stokes equations. LBM ability to efficiently simulate flows in problems with moving walls gives this method a great advantage in modeling the intrinsic pumping in lymphatic system [35]. In LBM, the time evolution of the distribution function of the fluid ‘particles’ is evaluated. The discretized distribution function ${f_i}\left( {r,t} \right)$ is integrated over a given time step through streaming and collisions of the fluid ‘particles’, where $i$, $r$, and $t$ are the lattice direction, position, and time. We use D3Q19 lattice with a 19-velocity distribution function in three spatial directions and a two relaxation time collision operator [39].

A lattice spring model (LSM) is implemented for modeling the vessel and elastic valves [40, 41], where the model components are represented by a planar network of springs connecting point masses. Stretching springs have a stiffness ${k_{\text{s}}}$ related to the Young’s modulus $E$ [41]. Bending springs with bending stiffness ${k_{\text{b}}}$ connect three collinear LSM nodes giving rise to bending rigidity ${d_{\text{b}}}$ [40]. The method of momentum exchange combined with the interpolated bounce-back rule is used to ensure momentum-conserving two-way coupling at the fluid-solid boundaries [37, 42]. We have previously extensively validated our FSI approach including simulations of lymphatic vessels [35, 43].

**Figure 1.**
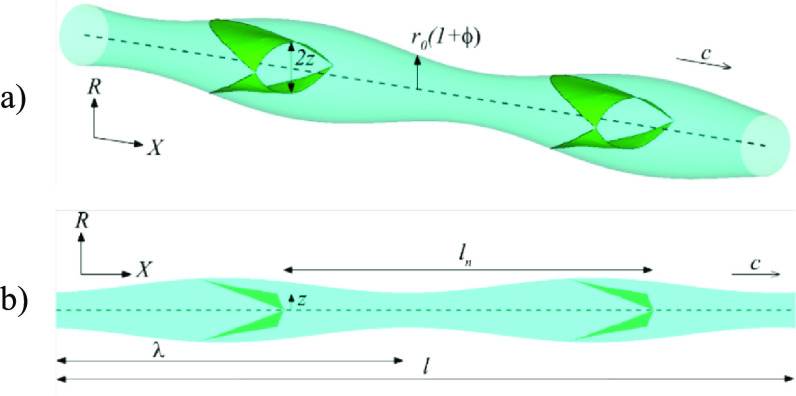
Model of a peristaltic vessel with two valves: (a) isometric view, (b) side view.

Our model of a lymphatic vessel with elastic valves is shown in figure 1 [35]. It is composed of a periodic fluid-filled axisymmetric vessel that undergoes peristaltic contractions given by ${r_{{\text{vess}}}}\left( {x,t} \right) = {r_0}\left[ {1 + \phi {\text{sin}}\left( {2\pi \left( {x - ct} \right)/\lambda } \right)} \right]$, where ${r_{{\text{vess}}}}$, ${r_0}$, $\phi $, $c$, $t$, and $\lambda $ are the vessel radius, mean vessel radius, normalized contraction amplitude, wave speed, time, and wavelength, respectively. The vessel is furnished with elastic valves. The valve leaflets are formed by an intersection between the vessel and a flat surface at an angle [34]. The leaflet is firmly attached to the vessel wall and follows its motion. The free edge of the leaflet has a semicircular cutout mimicking the typical geometry observed for lymphatic valves [44]. The leaflet aspect ratio is set to 1.75 that is in the typical range of experimental values [34].

The LBM domain has $44$ by $44$ grid nodes in the cross-sectional directions and a length ranging from $601$ to $901$ nodes depending on the vessel length. The number of LSM nodes range from 13 172 to 22 572 based on the vessel length and the number of valves. The average spacing between two neighboring LSM nodes is about twice the LBM grid spacing.

The radius of the vessel changes periodically in time as a function of the mean vessel radius ${r_0}$ and the contraction amplitude $a$ yielding a contraction wave that travels along the vessel [35]. Figure 1(b) shows a side view of a periodic vessel with two valves that are separated by a distance ${l_v} = l/{n_v}$, where ${n_v}$ is the number of valves and $l$ is the vessel length in the simulations. We consider ${l_v}$ in the wide range from $6{r_0}$ to $75{r_0}$ which is typical for lymphatic systems [45, 46].

The peristaltic wave travels with a constant wave speed $c = \lambda /\tau $, where $\tau $ is the contraction period. The wave speed $c$ defines the peristaltic Reynolds number ${\text{Re}} = \rho c{r_0}^2/\mu \lambda $, where $\rho $ is the fluid density and $\mu $ is the fluid dynamic viscosity [47]. We set ${\text{Re}} = 0.4$, which is representative for typical flow conditions in lymphatic vessels [1]. The normalized contraction amplitude of the vessel $\phi = a/{r_0}$ is set to 0.25 approximating the contraction amplitude of collecting lymphatics [48]. The normalized wavelength is $\Lambda = \lambda /2{r_0}$, and the normalized valve spacing is $L = {l_v}/\lambda $. In our simulations, $\Lambda $ is in the range from $5$ to $15$ consistent with experimental values [35], whereas $L$ is varied between 0.4 and 2. To characterize the valve opening, we use the dimensionless distance from the valve trailing edge to the vessel centerline $Z = z/{r_0}$ (figure 1(b)).

The magnitude of the adverse pressure gradient is normalized to represent a ratio between pressure and viscous forces $\Delta P = {p_x}\lambda r_0^2\rho /{\mu ^2}$, where ${p_x}$, $\rho $, $\mu $ are the adverse pressure gradient, fluid density, and fluid dynamic viscosity, respectively [49]. The values of the adverse pressure gradient tested in our study are $\Delta P = 0$, 70, 140, and 210.

In-plane and bending stiffnesses are normalized as ${K_{\text{s}}} = {k_{\text{s}}}\rho {r_0}/{\mu ^2}$ and ${K_{\text{b}}} = {k_{\text{b}}}\rho /{\mu ^2}{r_0}$, respectively, representing ratios between in-plane and bending forces and the viscous force. We set ${K_{\text{s}}} = 115$ and ${K_{\text{b}}} = 88$ [35]. Furthermore, the dimensionless axial coordinate is $X = x/\lambda $, radial coordinate is $R = r/{r_0}$, axial component of flow velocity is ${U_x} = {u_x}/c$, and time is $T = t/\tau $.

We evaluate the pumping performance of lymphatic vessels using period-averaged flow rate $q$ normalized as $Q = q/{q_0}$, where ${q_0} = \pi \!r_0^2c$ representing the flow rate in a rigid pipe of radius ${r_0}$ and mean flow velocity of $c$ [23, 24]. The period-averaged work done by the vessel wall $w$ is normalized as $W = w/\left( {8\mu \lambda \pi {c^2}\tau } \right)$, where the denominator represents viscous loss in a rigid pipe with the length of $\lambda $ and an average flow velocity of $c$. The pumping efficiency is $\eta = q{p_x}\lambda \tau /w$ and represents the ratio of the work applied to move the fluid against ${p_x}$ and the work done by the contracting vessel with the same wavelength. At least ${\text{five}}$ contraction cycles are simulated to reduce the effects of the initial transient, which ensures that the flow rate and work done by the vessel differs by less than $1{\% }$ from the preceding contraction cycle.

## 3. Results and discussions

### 3.1. Flow pattern

**Figure 2.**
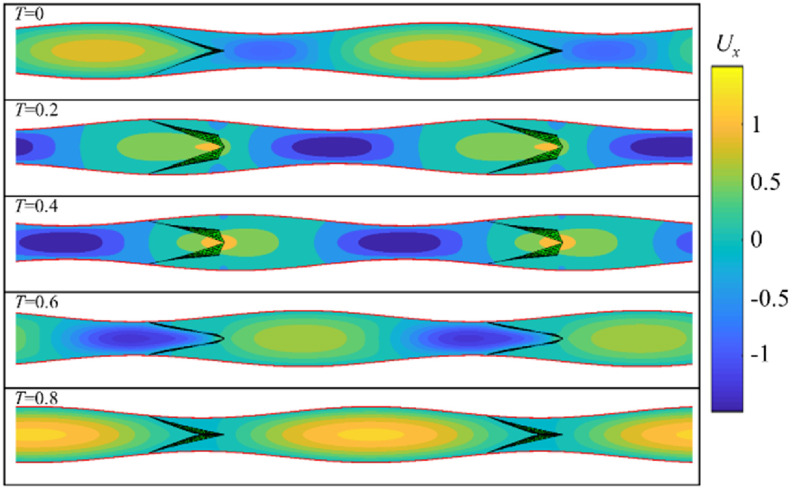
Axial flow velocity ${U_x}$ for $L = 1$, $\Lambda = 7.5$, $\Delta P = 140$, and ${\text{Re}} = 0.4$, under different phases of the contraction cycle $T$. See supplementary video S1.

**Figure 3.**
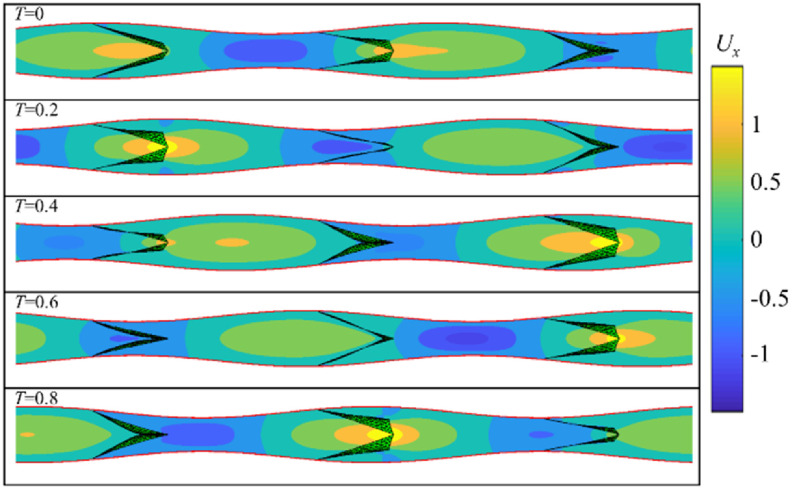
Snapshots of the axial flow velocity ${U_x}$ for vessel with $L = 2/3$, $\Lambda = 7.5$, $\Delta P = 140$, and ${\text{Re}} = 0.4$ at different phases of the contraction cycle $T$. See supplementary video S2.

Figures 2 and 3, respectively, show representative phases of the contraction cycle for the vessels with valves that are spaced equally to the contraction wavelength (i.e. $L = 1$), and with valves that are separated by a distance shorter than the wavelength (i.e. $L = 2/3$). As we discuss below, these values of $L$ correspond to the minimum and maximum pumping efficiencies of the vessel. The length of simulation domain equals to two contraction wavelengths and contains two and three valves for $L = 1$ and $L = 2/3$, respectively. The fluid flow and the valve dynamics repeat each cycle of the traveling wave. In figure 2, the valves open and close synchronous to each other. By contrast, the valves in figure 3 open and close with a time lag with respect to the neighboring valves leading to their overall asynchronous valve kinematics.

Periodic dilation and contraction of the vessel wall due to the propagation of a contraction wave result in the formation of positive and negative velocity packets traveling throughout the vessel. For $L = 1$, all the valves simultaneously encounter positive and negative velocity packets. For $L = 2/3$, at any given moment, different valves interact with the fluid with different flow velocities ranging from positive to negative depending on the valve position in the vessel.

### 3.2. Valve opening

**Figure 4.**
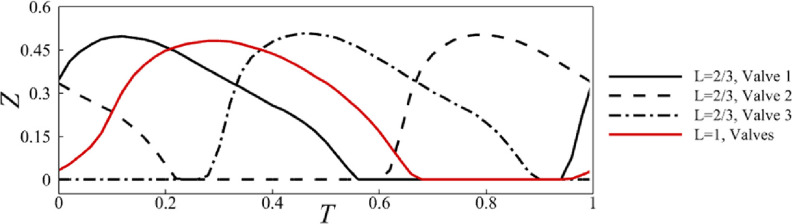
Displacement of valve trailing edge for vessels with $L = 2/3$ and $1$, $\Lambda = 7.5$, $\Delta P = 140$, and ${\text{Re}} = 0.4$ as a function of the contraction cycle $T$.

To analyze the valve opening behavior, we plot in figure 4 the displacement of the leaflet trailing edge as a function of contraction cycle for $L = 2/3$ and $L = 1$. All valves exhibit similar trailing edge kinematics. Unlike the synchronous valves that open and close simultaneously, there is a time lag of $2T/3$ between consecutive valves in the case of $L = 2/3$. For $L = 2/3$, the valves are open about 64% of their period, whereas for $L = 1$, the valves are open for 62% of their period. Thus, the asynchronous valves remain open for a slightly longer period than synchronous valves.

### 3.3. Centerline axial velocity

**Figure 5.**
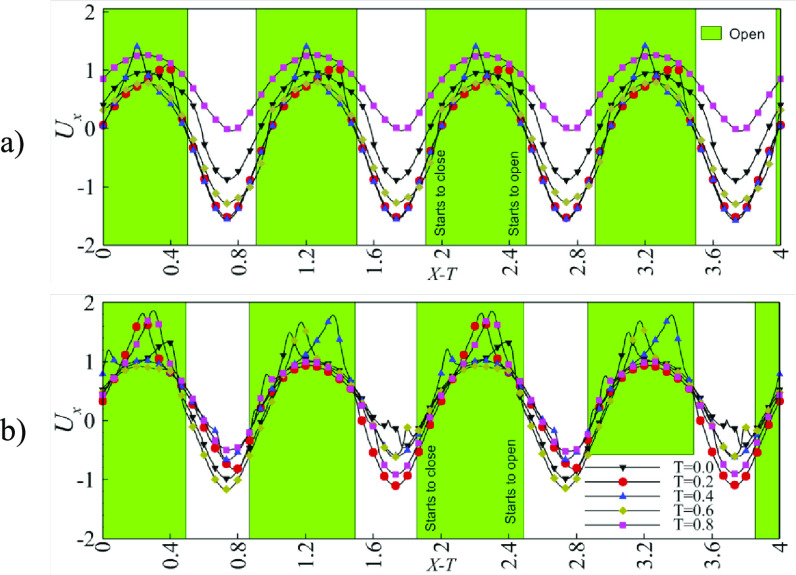
(a) Centerline axial velocity ${U_x}\left( 0 \right)$ for vessel with $L = 1$, $\Lambda = 7.5$, $\Delta P = 140$, and ${\text{Re}} = 0.4$. (b) Centerline axial velocity ${U_x}\left( 0 \right)$ for $L = 2/3$, $\Lambda = 7.5$, $\Delta P = 140$, and ${\text{Re}} = 0.4$. The centerline velocities are plotted in a moving frame of reference $X - T$. The green highlighted areas indicate the instances at which the valves are open.

Figures 5(a) and (b) show the centerline axial velocities for vessels with $L = 1$ and $L = 2/3$, respectively, for different phases of the contraction cycle. The velocities are plotted in the reference frame moving with the speed of the vessel contraction wave. In this reference frame, the flow velocity of a peristaltic vessel is steady [35]. Highlighted sections indicate the instances when the valves are open. In the case of $L = 1$, the periodicity of the centerline velocity is equal to $T$; however, this value is equal to $2T$ for $L = 2/3$.

Peaks of the centerline velocity correspond to the instances when the fluid accelerates as it moves through the orifice of a partially open valve. We find that for asynchronous valves the flow velocity fluctuates more strongly compared to the synchronous valves indicating a faster flow velocity. We find that the opening and closing events for all valves overlap when presented in the moving reference frame $X - T$. This occurs for the vessels with synchronous and asynchronous valves highlighting the periodicity of the valve motion. Consistent with figure 4, the valves with $L = 2/3$ remain open longer than valves with $L = 1$, as evident by the wider width of the green area in figure 5. The valve opening occurs at about the same $X - T$ for synchronous and asynchronous valves; however, valve closing is delayed for asynchronous valves leading to the overall longer open phase.

### 3.4. Pumping performance

**Figure 6.**
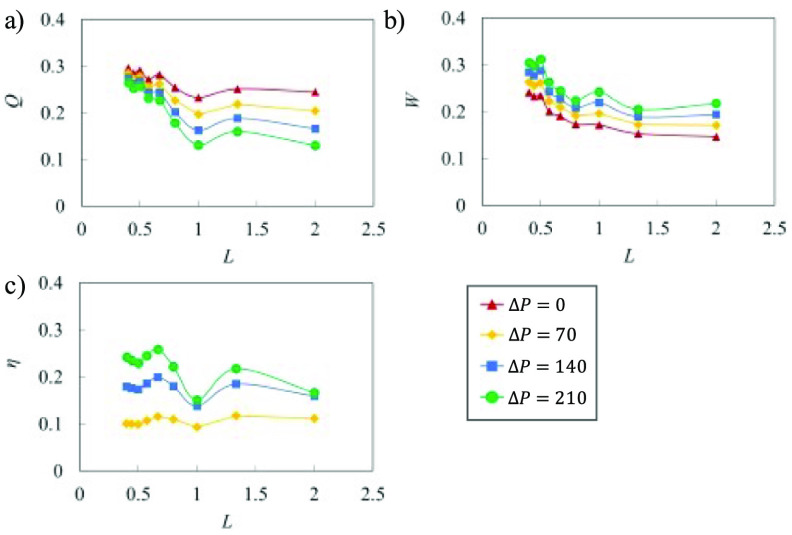
(a) Flow rate, (b) work done by the vessel, and (c) pumping efficiency as a function of normalized valve spacing $L$ for vessels with $\Lambda = 7.5$ and ${\text{Re}} = 0.4$.

The dependence of the pumping performance on the valve spacing is presented in figure 6 for different values of the adverse pressure gradient $\Delta P$. We find that the flow rate $Q$ is maximized when $L &lt; 0.5$ (figure 6(a)) indicating that asynchronous valves enhance pumping flow rate. Since the adverse pressure gradient $\Delta P$ acts against the positive flow direction, increase in $\Delta P$ results in lower $Q$. While $Q$ drops with increasing $\Delta P$, the difference between different $\Delta P$ is less significant when $L$ decreases to about 0.5. This suggests that valves with lower spacing are beneficial for reducing back flow emerging due to an adverse pressure gradient.

For a given $\Delta P$, $Q$ shows noticeable drop for synchronous valves with $L = 1$ and $L = 2$, indicating a subpar pumping when valve spacing is integer multiple of the contraction wavelength. Furthermore, the difference between $Q$ for $L = 1$ and $L = 2$ is insignificant, despite the vessel with $L = 1$ has twice more valves than with $L = 2$. In fact, the vessel with $L = 2$ slightly outperforms the vessel with $L = 1$ due to lower flow resistance caused by the valves. As it can be seen in the figure 6(b), lower values of $L$ require greater work $W$ for pumping the fluid. We relate this increase in $W$ to the higher number of valves per unit vessel length that increase the flow resistance.

Efficiency of the vessel pumping $\eta $ is presented in figure 6(c). Note that we do not show $\eta $ for $\Delta P = 0$ since our definition of efficiency has $\Delta P$ in the nominator. The simulations show that asynchronous valves yield higher efficiency than synchronous valves. Thus, the benefit of increased pumping flow rate outweighs the negative impact of higher work demand. The efficiency is maximized around $L = 2/3$ where the balance between $Q$ and $W$ is optimal. For $L &lt; 2/3$, the efficiency $\eta $ decreases indicating that too short valve spacing is detrimental for the overall pumping efficiency.

**Figure 7.**
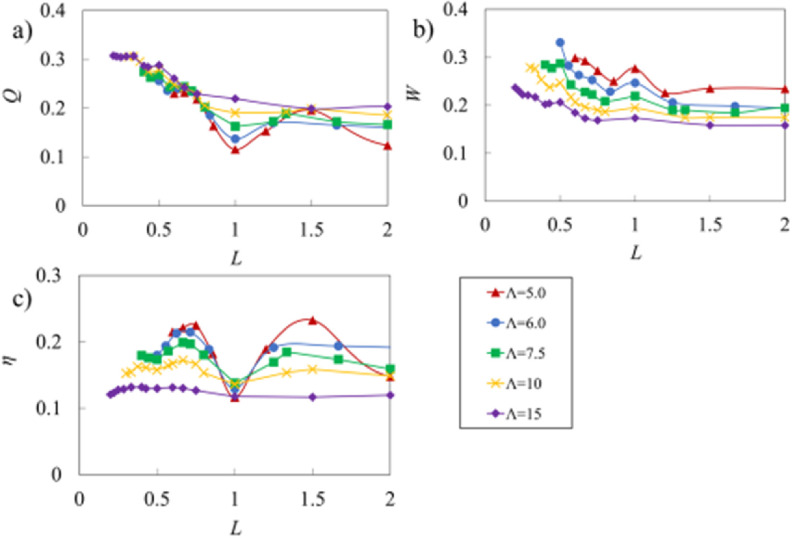
(a) Flow rate, (b) vessel work, and (c) pumping efficiency as a function of normalized valve spacing $L$ for different $\Lambda $ for vessels with ${p_x} = 9$ and ${\text{Re}} = 0.4$.

In figure 7, we show the dependence of the vessel pumping on $L$ for different wavelength $\Lambda $. Here, we set ${p_x} = 9$. Note that since $\Delta P$ depends on $\Lambda $, the values of $\Delta P$ in this figure change with $\Lambda $. Furthermore, we keep the contraction period $\tau $ equal for all these simulations, leading to the contraction wave speed $c$ increasing with increasing $\Lambda $. This, in turn, results in the normalization for $Q$ and $W$ changing by $\Lambda $ and ${\Lambda ^2}$, respectively.

We find that $Q$ is insensitive to $\Lambda $ when $L &lt; 2/3$ as shown in figure 7(a), meaning that in this case $q$ is proportional to $c$. The difference among different $\Lambda $ is the most significant for synchronous valves with $L = 1$ and $L = 2$, in which case the flowrate increases with $\Lambda $. This result suggests that $q$ increases faster than $c$, meaning that for lower $\Lambda $ the synchronous valves are less efficient in preventing the back flow.

Figure 7(b) shows that the dimensionless work $W$ decreases with increasing $\Lambda $, since greater $\Lambda $ results in fewer valves per unit vessel length and, therefore, the relative contribution of viscous losses at the valves decreases. For $\Lambda = 15$, $W$ only slightly increases with decreasing $L$, since the work is mostly required to overcome viscous losses arising along the vessel walls. Note that since $W$ is normalized using ${\Lambda ^2}$, dimensional work $w$ increases with $\Lambda $.

Pumping efficiency $\eta $ for different values of $\Lambda $ is shown in figure 7(c). The efficiency is the lowest for $\Lambda = 15$ and is nearly independent of $L$. We attribute it to the significant viscous losses in long vessels. The efficiency $\eta $ remains low for all $\Lambda $ when the valves operate synchronously. Asynchronous valves on the other hand yield significantly higher efficiency.

### 3.5. Volumetric pumping

**Figure 8.**
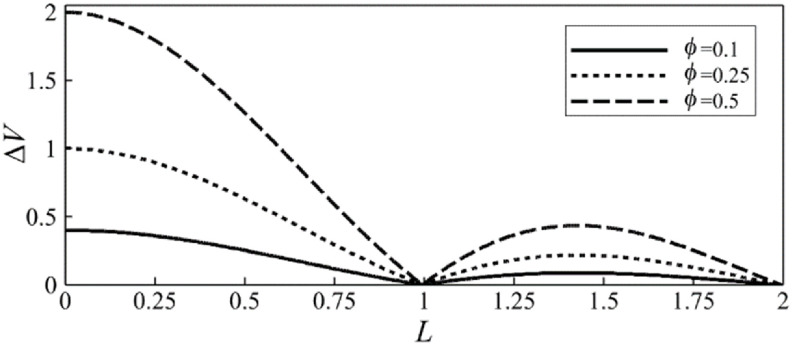
Normalized lymphangion volume change $\Delta V$ as a function of valve spacing $L$ for different contraction amplitudes $\phi $, $\Lambda = 7.5$, $\Delta P = 140$, and ${\text{Re}} = 0.4$.

To rationalize the beneficial effects of the asynchronous valve opening on the lymphatic pumping we evaluate the volume change of an individual lymphangion during a vessel contraction period. In figure 8, we show the lymphangion volume change $\Delta V = \left( {{v_{{\text{max}}}} - {v_{{\text{min}}}}} \right)/{v_0}$ as a function of $L$. Here, ${v_{{\text{max}}}}$ and ${v_{{\text{min}}}}$ are respectively the maximum and minimum volume of a lymphangion during a contraction period, and ${v_0} = \pi \!r_0^2{l_v}$ is the mean lymphangion volume. The figure show that the volume change $\Delta V$ is zero when $L = 1$ and $L = 2$, i.e. the valves operate synchronously, indicating that in these cases the volume of the lymphangions remains constant during the vessel periodic contractions. For asynchronous valves $\Delta V$ is greater than zero, correlating well with the increased pumping rate for such valves in figures 6(a) and 7(a). Furthermore, $\Delta V$ is independent of the wavelength $\Lambda $ and the adverse pressure gradient, and only changes with the wave amplitude $\phi $. These results suggest that lymphangion volume change facilitates the vessel fluid pumping. Furthermore, pumping is maximized when $L &lt; 1$, suggesting that optimal pumping occurs when the contraction wavelength is longer than the distance between valves.

## 4. Summary

Using computational modeling, we investigate fluid pumping against an adverse pressure gradient in a peristaltic vessel fitted with elastic unidirectional valves. We considered vessels with different valve spacing relative to the vessel contraction wavelength. When the valve spacing equals to the contraction wavelength, the valves open and close synchronously. A difference between the valve spacing, and the wavelength can lead to asynchronous valve openings with a phase difference related to the ratio of the valve spacing and the wavelength. We find that asynchronous valves are open for extended fraction of their period compared to the synchronous valves. Furthermore, we find that asynchronous valves lead to greater pumping performance and higher efficiency for a range of adverse pressure gradients and contraction wavelengths. We relate the superior pumping performance of vessels with asynchronous valves to the cyclic volume change of lymphangions that connect consecutive one-way valves. The volume change enables unidirectional flow in the lymphatic vessel that compliments the flow induced by the traveling contraction wave. The results of our study help to rationalize the effects of valve spacing on the operation of lymphatic system and to better understand the potential impacts of pathological conditions leading to abnormal contraction wave pattern.

Furthermore, our results provide guidelines for designing synthetic pumping systems that can mimic the operation of lymphatic vessels. Peristaltic micropumps are widely used in lab-on-a-chip devices and drug delivery systems [50]. These miniature flexible pumps fabricated from biocompatible materials are especially attractive for *in-vivo* applications, where higher pumping efficiency and reduced power consumptions are important considerations. In this regard, the results of our study are useful for developing peristaltic micropumps furnished with elastic valves that mimic the operation of lymphatic vessels. Incorporating valves that prevent backflow enables superior pumping performance compared to valveless peristaltic vessels especially when the pumping is against an adverse pressure gradient [35]. Our results indicate the pathway for optimizing the pumping performance by adjusting the spacing between the valves, thereby enabling the design of efficient miniature pumping devices.

## Data Availability

All data that support the findings of this study are included within the article (and any supplementary files).
